# The College and the Hospital

**Published:** 1883-09

**Authors:** 


### The College and the Hospital.

In the last number of the Journal we had occasion to refer to recent changes in the *personnel* of the faculty of the Buffalo Medical College, and also to the controlling influence which that institution exerts in appointments on the medical and surgical staff of the hospital. In what we said, we reflected the current sentiment of the profession in this city in regard to the undue preponderance of college influence in the formation of that staff. If doubt has heretofore existed in the minds of any as to the status of affairs at the hospital, the appointment of the new lecturer on surgery, a young man unknown to the trustees, unknown to the staff by whom he was recommended, and almost unknown to fame, to a vacant position on the surgical staff, simply because the college wished to make use of the hospital for the purposes of clinical instruction for its classes, is amply sufficient to remove it. This appointment, in disregard of the claims of our local surgeons, confirms all we have asserted, that the hospital, is used by the college merely as an appendage to the latter, and to subserve its selfish purposes. Allow us to state, in this connection, that the use of patients for the clinical instruction of students in medicine is not a subject for unfavorable criticism. We simply contend here that the profession of the city should receive deserved recognition in hospital appointments. At the dictation of the college, the trustees have entirely ignored this principle, and have conferred its honors, if such they be termed, upon one who has no claim whatever upon it, except such as is derived through his connection with the college. If the hospital, after showing this disrespect to the profession of the city, expects its co-operation and sympathy in the support and maintenance of that institution, we think it is reckoning on a larger measure of forbearance than human nature is wont to manifest under like circumstances. This action simply indicates that the hospital authorities care more for the "coterie of medical men" who constitute the faculty of the college than for the entire profession of the city. Let the profession make a note of this. Have they patronage to confer upon an institution which imports, from another city, a surgeon to treat their patients? Have we not local talent equal to all the emergencies that may arise, without sending to Chicago for aid? Not a flattering compliment to our ability when it is announced in the local press that we have not a surgeon "equal to all the varied requirements" of a position in the college, *ergo* not in the hospital.

It is not our custom to notice strictures upon our opinions published in the secular press, but an anonymous writer in one of our city dailies, in a feeble and ill-advised effort to reply to the criticisms in which we indulged on the college and hospital, in our last number, has placed at our disposal so frank a confession of the lamentable failure of the college to fulfill its duty to the profession and to the public that we cannot forbear to notice it. What a commentary upon the reputation and success of a college when it acknowledges, in the public press, that after over forty years of labor in educating men in medical science, "there is no one in Buffalo," not even among its alumni, "equal to the varied requirements" of a position in its faculty. The appeal to the honest opinion of the medical profession of this city, to sustain so glaring an insult to the professional attainments, experience and skill of its mem bers, is the effort of a weak man to hide himself behind the shadow of his neighbors.

In what we have said, we disclaim any discourtesy to the new lecturer on surgery, or any intention to disparage his reputation or ability. While not offering any apology for the strong position we have felt ourselves called upon, in the interests of the profession of this city, to assume, we can only say that inasmuch as he has voluntarily chosen unpopular professional associations, he must bear up under the odium and prejudice to which such associations give rise.

